# Supplementary material for: Curated and harmonised transcriptomics datasets of interstitial lung diseases
Source: Data Brief. 2025 Oct 14;63:112139. doi: 10.1016/j.dib.2025.112139 (PMC12581653; doi:10.1016/j.dib.2025.112139)
Supplement: Supplementary file 1 [file mmc1.zip › Supplementary_material/RNA-seq/fastQC reports/GSE213001/SAMN30751059_fastqc.html]

SAMN30751059.fastq.gz FastQC Report 

FastQC Report

Wed 27 Sep 2023  
SAMN30751059.fastq.gz

## Summary

- Basic Statistics
- Per base sequence quality
- Per tile sequence quality
- Per sequence quality scores
- Per base sequence content
- Per sequence GC content
- Per base N content
- Sequence Length Distribution
- Sequence Duplication Levels
- Overrepresented sequences
- Adapter Content

## Basic Statistics

| Measure | Value |
| --- | --- |
| Filename | SAMN30751059.fastq.gz |
| File type | Conventional base calls |
| Encoding | Sanger / Illumina 1.9 |
| Total Sequences | 18309849 |
| Sequences flagged as poor quality | 0 |
| Sequence length | 100 |
| %GC | 46 |

## Per base sequence quality

## Per tile sequence quality

## Per sequence quality scores

## Per base sequence content

## Per sequence GC content

## Per base N content

## Sequence Length Distribution

## Sequence Duplication Levels

## Overrepresented sequences

| Sequence | Count | Percentage | Possible Source |
| --- | --- | --- | --- |
| ATCGGAAGAGCACACGTCTGAACTCCAGTCACCATAATACATCTCGTATG | 23432 | 0.12797484020758446 | TruSeq Adapter, Index 7 (97% over 36bp) |
| GATCGGAAGAGCACACGTCTGAACTCCAGTCACCATAATACATCTCGTAT | 20652 | 0.11279175486373483 | TruSeq Adapter, Index 7 (97% over 37bp) |
| CCCGTATCGAAGGCCTTTTTGGACAGGTGGTGTGTGGTGGCCTTGGTATG | 19311 | 0.10546782772484907 | No Hit |
| CTTGGATTAAGGCGACAGCGATTTCTAGGATAGTCAGTAGAATTAGAATT | 19019 | 0.1038730576095958 | No Hit |
| GGCGGATGAAGCAGATAGTGAGGAAAGTTGAGCCAATAATGACGTGAAGC | 19004 | 0.1037911344872369 | No Hit |
| GGCAAATACAGCTCCTATTGATAGGACATAGTGGAAGTGGGCTACAACGT | 18939 | 0.10343613429034833 | No Hit |

## Adapter Content

Produced by FastQC (version 0.11.7)
